# The soluble pyocins S2 and S4 from *Pseudomonas aeruginosa* bind to the same FpvAI receptor

**DOI:** 10.1002/mbo3.27

**Published:** 2012-06-22

**Authors:** Ameer Elfarash, Qing Wei, Pierre Cornelis

**Affiliations:** Department of Bioengineering Sciences, Research Group of Microbiology, VIB Department of Structural Biology, Vrije Universiteit BrusselPleinlaan 2, B-1050, Brussels, Belgium

**Keywords:** Colicins, iron homeostasis, *Pseudomonas aeruginosa*, siderophores

## Abstract

Soluble (S-type) pyocins are *Pseudomonas aeruginosa* bacteriocins that kill nonimmune *P. aeruginosa* cells by gaining entry via a specific receptor, which, in the case of pyocin S2, is the siderophore pyoverdine receptor FpvAI, and in the case of pyocin S3, FpvAII. The nucleic acid sequence at the positions 4327697–4327359 of *P. aeruginosa* PAO1 genome was not annotated, but it was predicted to encode the immunity gene of the flanking pyocin S4 gene (PA3866) based on our analysis of the genome sequence. Using RT-PCR, the expression of the immunity gene was detected, confirming the existence of an immunity gene overlapping the S4 pyocin gene. The PA3866 coding for pyocin S4 and the downstream gene coding for the immunity protein were cloned and expressed in *Escherichia coli* and the His-tagged S4 pyocin was obtained in pure form. Forty-three *P. aeruginosa* strains were typed via PCR to identify their ferripyoverdine receptor gene (*fpvA*I–III) and were tested for their sensitivity to pyocin S4. All S4-sensitive strains had the type I ferripyoverdine receptor *fpvA* gene. Some S4-resistant type I *fpvA*-positive strains were detected, but all of them had the S4 immunity gene, and, following the deletion of the immunity gene, became S4-sensitive. The *fpvAI* receptor gene was deleted in a S4-sensitive strain, and, as expected, the mutant became resistant to S4. The *N*-terminal receptor binding domain (RBD) of pyocin S2, which also uses the FpvAI receptor to enter the cell, was cloned in the pET-15b vector, and expressed in *E. coli*. When the purified RBD was mixed with pyocin S4 at different ratios, an inhibition of killing was observed, indicating that S2 RBD competes with the pyocin S4 for the binding to the FpvAI receptor. The S2 RBD was also shown to enhance the expression of the *pvdA* pyoverdine gene, suggesting that it, like pyoverdine, works via the known siderophore-mediated signalization pathway.

## Introduction

One of the strategies used by bacteria to protect themselves against close relative competitors is the production of antibacterial proteins, called bacteriocins (Baba and Schneewind 1998; Riley 1998; Riley and Wertz 2002). Over 90% of the *Pseudomonas aeruginosa* strains produce pyocins, which kill bacteria of the same species (Michel-Briand and Baysse 2002). Pyocins are divided into two groups: the R- and F-type pyocins, which are insoluble and kill bacteria by depolarization of the cytoplasmic membrane, and the S-type pyocins, which are soluble and protease-sensitive (Michel-Briand and Baysse 2002). Soluble pyocins also differ in their way of killing target cells, which can be via a DNase, tRNase, or pore-forming activity (Michel-Briand and Baysse 2002; Ling et al. 2010).

S-pyocins have four domains: (I) a receptor binding domain (RBD), (II) a domain with a still unknown function, (III) a translocation domain, and (IV) a killing domain, and are encoded by one open reading frame (ORF). A second ORF encodes an immunity protein that binds and inhibits the killing domain of the protein to protect the producing cells from their own pyocin-activity (Michel-Briand and Baysse 2002). S-pyocins also differ in their RBD, whereas the nuclease killing domain is more conserved (Sano 1993; Duport et al. 1995; Kageyama et al. 1996).

When grown under conditions of iron limitation, *P. aeruginosa* produces the fluorescent pyoverdine molecule, which serves as a high-affinity siderophore (Ravel and Cornelis 2003; Visca et al. 2007; Cornelis 2010). Pyoverdines differ by the peptide chain attached to a conserved chromophore, and three different types of pyoverdines have been found to be produced by different *P. aeruginosa* strains (Cornelis 2010). Each pyoverdine type is recognized by a specific outer membrane receptor, FpvA I, II, or III (Bodilis et al. 2009). As the sequences of the three different types of *fpvA* genes are known, it is possible to easily determine which *fpvA* receptor gene is present in the genome of a given strain (de Chial et al. 2003; Ghysels et al. 2004; Bodilis et al. 2009). Ohkawa et al. (1980) first reported that mutants resistant to S2 pyocin fail to produce an iron-repressed outer membrane protein, but did not establish the link with the ferripyoverdine receptor. Smith et al. (1992) suggested that the pyocin Sa receptor could be the ferripyoverdine receptor. Later on, it was found that the receptor for type II ferripyoverdine, FpvAII, is used by S3 to kill sensitive *P. aeruginosa* strains (Baysse et al. 1999; de Chial et al. 2003). More recently, we confirmed that pyocin Sa is in fact pyocin S2 and that type I FpvA (the major type I ferripyoverdine receptor) is the receptor for this pyocin (Denayer et al. 2007). In the genome of *P. aeruginosa* PAO1 (http://www.pseudomonas.com), three loci encode S-type pyocins: PA1150–1151 (pyocin S2 and immunity gene), pyocin S4 (PA3866), and PA0984–0985 (immunity gene and pyocin S5 gene). Here, we show that the pyocin S4 gene is followed by an overlapping immunity gene, which is not annotated in the genome. We also demonstrate that purified S4 pyocin recognizes the same FpvA I receptor as S2, and that the RBD of S2 inhibits killing by S4.

## Materials and Methods

### Strains and plasmids

Bacterial strains and plasmids used in this study are described in Table 1. Bacteria were grown at 37°C in rich Luria broth (LB) medium (Life Technologies, Merelbeke, Belgium) or in iron-poor Casamino Acids (CAA) medium (Difco Laboratories, Detroit, Michigan), and cultures were shaken in a New Brunswick Innova 4000 shaker at 200 rpm.

**Table 1:** Strains and vectors used in this study

Strains or plasmids	Features	References /sources
***Pseudomonas aeruginosa***		
PAO1	Wild-type *P. aeruginosa*	Stover et al. 2000
ΔS4+imm	Chromosomal deletion mutant of pyocin S4 (PA3866) and immunity mutant in PAO1	This study
W15Aug30	Wild-type *P. aeruginosa* sensitive to pyocin S4	Denayer et al. 2007
W15Aug30Δ*fpvAI*	Chromosomal deletion mutant of *fpvA I* (PA2398) mutant in W15Aug30	This study
***Escherichia coli***		
BL21(DE3)	F^–^ *ompT gal dcm lon hsdS*_B_(r_B_^−^ m_B_^−^) λ(DE3 [*lacI lacUV*5-T7 gene 1 *ind*1 *sam*7 *nin*5])	Studier et al. 1990
S17-1 *λpir*	*thi pro hsdR hsdM+ recA* RP4-2 Tc::Mu-Km::Tn*7 pir*, used for conjugation or biparental mating	de Lorenzo and Timmis 1994
**Plasmids**		
pDM4	Suicide vector carrying *sacBR* genes for sucrose sensitivity, Cm^r^	Milton et al. 1996
pDMS4imm	pDM4 containing the two flanking fragments of PA3866+imm, Cm^r^	This study
pDMfpvA1	pDM4 containing the two flanking fragments of PA2398, Cm^r^	This study
pET15b	Expression vector, N-terminal his-tag, Ap^r^	Studier et al. 1990
pETS4imm	pET15b with coding sequence for PA3866 gene and the immunity gene, Ap^r^	This study
pETRBD	pET15b with the first 648 bp coding sequence of PA1150 gene, Ap^r^	This study
pRK2013	Mob^+^, Tra^+^, ColE1, mobilization vector, Km^R^	Figurski and Helinski 1979

Ap^r^, Km^r^, Cm^r^, Tc^r^, and Gm^r^ indicate resistance to ampicillin, kanamycin, chloramphenicol, tetracycline, and gentamycin, respectively.

### RNA isolation and RT-PCR

Bacterial cells were harvested in stationary phase and bacterial RNA was extracted using the High Pure RNA Isolation Kit (Roche, Vilvoorde, Belgium). The purity and concentration of the RNA was determined by gel electrophoresis and spectrophotometry (NanoDrop, Ijsselstein, Netherlands). First-strand cDNA was reverse transcribed from one microgram of total RNA using First-strand cDNA Synthesis Kit (Amersham Biosciences, GE Healthcare, Diegem, Belgium). RT-PCR was performed using primers (S4imf and S4imr) described in Table 1.

### Quantitative real-time PCR (qRT-PCR)

qRT-PCR was performed in a Bio-Rad iCycler with Bio-Rad iQ SYBR Green Supermix. For all primer sets (Table 2), the following cycling parameters were used: 94°C for 3 min followed by 40 cycles of 94°C for 60 s, 55°C for 45 s, and 72°C for 60 s, followed by 72°C for 7 min. *oprI* (house-keeping gene control, outer membrane lipoprotein precursor) was used to normalize gene expression.

**Table 2:** List of primers used in this study

Name	Primer sequence (5'→3')	RE
**Amplification of S4 immunity gene**		
S4imf	AGGCAATGGGAAGATGTGG	
S4imr	CCTCTGTACTCTCTTTCGC	
**Pyocin S4 cloning primers**		
CS4f	GGAATTCCATATGACAAATAATAGTGCGCCACCAC	*Nde1*
CS4r	CCGCTCGAGTTATTTTCTGGAGGCAATTGTTAC	*XhoI*
**S4+imm** (PA3866**+imm) deletion primers**		
DelS4fu	GCGTCGACGTGGATGTGGTGTCGATGTTC	*Sal I*
DelS4ru	AGTCCATGCAAGGGAGAATGGCTTTCCTATCGAAAGAG	
DelS4fd	ATAGGAAAGCCATTCTCCCTTGCATGGACTACAAAC	
DelS4rd	GCTCTAGACCATTTCAGCTATCACGGTTA	*Xba I*
***fpvA1* (**PA2398**) deletion primers**		
*fpvA*fu	CGGGATCCCTATTCGACGACCTGGTCCA	*BamHI*
*fpvA*ru	GAACATCAGGTTCCGACCGTGTGGTGCTGGCATGG	
*fpvA*fd	CCAGCACCACACGGTCGGAACCTGATGTTCAGCAC	
*fpvA*rd	GCTCTAGAGAAATCGCACAGAGCAACG	*Xba I*
**RBD of S2 gene (**PA1150**) cloning primers**		
CRBDf	GGAATTCCATATGGCTGTCAATGATTACGAACCTG	*Nde1*
CRBDr	CCGCTCGAGCTCGACATTTGCCTTCCTGG	*Xho1*
**Real Time PCR primers**		
*pvdA*FWD	CACAGCCAGTACCTGGAACA	
*pvdA*REV	GGGTAGCTGTCGTTGAGGTC	
*oprI*FWD	ATGAACAACGTTCTGAAATTCTCTGCT	
*oprI*REV	CTTGCGGCTGGCTTTTTCCAG	

The underlined sequences, added to the primer fragments, indicates the recognition sites of the restriction enzyme (RE) or the overlapping sequence used for the fusion.

### Overexpression and purification of pyocin S4 and the RBD of pyocin S2

Pyocin S4 gene with the immunity gene (pyoS4+im, PA3386, 2633 bp) and the RBD of pyocin S2 (RBD, first 648 bp of PA1150) were cloned from PAO1 using primers listed in Table 2. Amplified fragments were introduced into pET15b (+) (Merck, Germany) by *Nde*I/*Xho*I double digestion, ligation, and transformation into *Escherichia coli* BL21 (DE3) pLysS.

For overexpression, the transformants with the recombinant plasmids were induced for expression of the cloned gene by growing them overnight at 28°C in the presence of 1.0 mmol/L IPTG after the OD600 reached 0.7. The harvested cells were resuspended in TGE buffer (50 mmol/L Tris-HCl (pH 7.5), 10% glycerol, 1 mmol/L EDTA, and 10 mmol/L imidazole) and disrupted by pulsed sonication. The clear lysate was centrifuged at 20,000 rpm for 15 min, and the clear supernatant was loaded onto a Hi-Trap FF column (Amersham Biosciences, GE Healthcare) integrated by AKTA TM FPLC system (Amersham Biosciences, GE Healthcare). The His-tagged proteins were eluted using the elution buffer containing 1 mol/L imidazole in 20 mmol/L Tris-HCl (pH 7.5). The purity of the His-tagged proteins was verified as >95% homogeneity after 10% SDS polyacrylamide gel electrophoresis (SDS-PAGE, Invitrogen, Gent, Belgium). The purified proteins were dialyzed against 20 mmol/L Tris-HCl (pH 7.5) and 150 mmol/L NaCl. The protein concentration was determined using a NanoDrop 1000 spectrophotometer (Thermo Scientific, Doorveld, Belgium). The pooled pure proteins were divided into small aliquots and stored at −20°C, which were frozen and thawed individually before each manipulation.

### Pyocin sensitivity assays

To check the sensitivities of *P. aeruginosa* strains to pyocin S4, 10 μL of pyocin lysate was spotted onto a bacterial cell layer containing 5 × 10^6^ cells mL^−1^ and incubated at 37°C for 24 h.

### Strain and plasmid construction

Deletion mutants were constructed by allelic exchange as described by Milton et al. (1996). In short, DNA regions flanking the gene to be deleted were amplified by PCR using primers listed in Table 2, fused in a second PCR, and ligated into the suicide vector pDM4 (Milton et al. 1996). To introduce this plasmid into *P. aeruginosa*, triparental matings were done with the donor strain *E. coli* S17-1λpir containing the deletion constructs and a helper strain having pRK2013 as a mobilizing plasmid (Figurski and Helinski 1979). Transconjugants were selected on LB containing chloramphenicol. PCR analysis confirmed that the vector had integrated correctly into the chromosomal DNA. To complete the allelic exchange, the integrated suicide plasmid was forced to recombine out of the chromosome by adding 10% sucrose for several generations. The pDM4 vector contains the lethal *sacB* gene, which encodes a levansucrase and surviving colonies on LB plates with 10% sucrose were chosen, and the correct deletion was confirmed by PCR.

## Results

### Evidence for a pyocin S4 immunity gene

**Figure 1:**
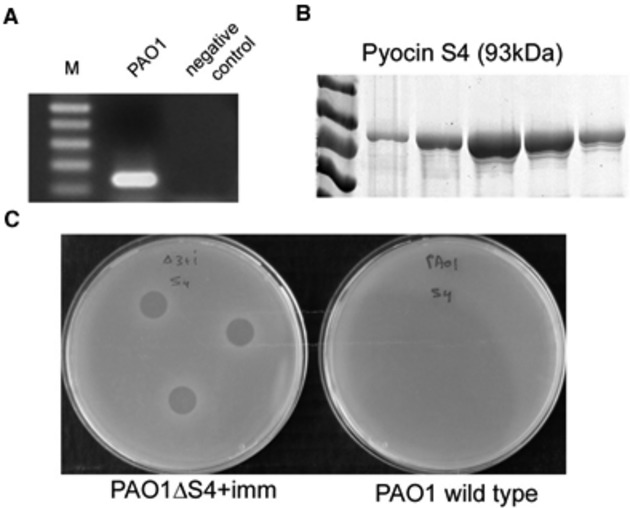
(A) Agarose electrophoresis of the RT-PCR reaction product to check for the expression of the S4 immunity gene. The figure confirmed the expression of the immunity gene in PAO1 (lane 2), whereas there was no amplification in the negative control containing only RNA (lane 3). (B) SDS–PAGE analysis of different purification fraction of pyocin S4 protein (93 kDa). (C) Pyocin S4 sensitivity of wild-type PAO1 (right) and PAO1 Δ3866+*imm* (left). PAO1 wild-type is immune and became sensitive after the deletion of the pyocin and immunity genes.

S-type pyocin producing cells have to protect themselves against the activity of their own pyocin. Free pyocins in the cytoplasm would kill the producing strain because they act as antibacterial effectors. Therefore, immunity proteins are produced that inhibit their cognate toxin by direct interaction (Michel-Briand and Baysse 2002). The immunity genes are tightly linked to the cognate pyocin genes encoding the killing proteins, and their expression is translationally coupled (Sano 1993; Sano and Kageyama 1993; Sano et al. 1993). But, in *P. aeruginosa* PAO1, no immunity gene for pyocin S4 was annotated in the genome database (http://www.pseudomonas.com), although it is predicted to be located next to the pyocin gene (PA3866). Therefore, RNA from *P. aeruginosa* PAO1 was isolated and reverse-transcribed to cDNA. The obtained cDNA was used as a template to perform a RT-PCR to check for the expression of the immunity gene. Our results (Fig. 1A) confirmed the expression of a gene downstream of the (PA3866) S4 gene, revealing the existence of the immunity gene overlapping the pyocin gene at the positions 4327697–4327359 of *P. aeruginosa* PAO1 genome. The stop codon of the S4 gene (TAA) overlaps by one base with the start codon of S4-imm ORF (ATG). The region comprising the pyocin S4 gene together with the immunity gene (pyoS4+imm, 2633 bp) was PCR-amplified from PAO1 DNA using primers listed in Table 2. The amplified fragment was introduced into the pET15b (+) vector (Merck, Germany) by *Nde*I/*Xho*I double digestion, ligation, and transformation into *E. coli* BL21 (DE3) pLysS. After induction and purification of the His-tagged protein, a protein of 93 kDa was detected on SDS-PAGE gels (Fig. 1B). The purified protein was found to be active as it caused the death of a sensitive *P. aeruginosa* strain (results not shown).

We later deleted both the pyocin S4 gene (PA3866) and the downstream immunity gene in PAO1 (PAO1ΔS4+imm), and then tested the sensitivity of this mutant to the purified pyocin S4. The results (Fig. 1C) showed that PAO1ΔS4+imm became sensitive to S4 after the deletion of the immunity gene, which confirms its function in the immunity against S4. It also suggests that PAO1 produces the receptor for pyocin S4.

### S4 pyocin, like S2, uses the FpvAI ferripyoverdine type I receptor to gain entry into the cells

To determine which receptor serves as the entry point of pyocin S4, a sensitivity assay was performed on 43 environmental strains recovered from the Woluwe river in order first to determine which ferripyoverdine receptor gene (*fpvA*I, II or III) is present in their genome (Pirnay et al. 2005; Denayer et al. 2007; Bodilis et al. 2009).

The results, which are presented in Table 3 reveal that all S4-sensitive strains (six strains) had the *fpvAI* receptor gene, whereas the 19 strains having the two other types of receptor genes were all insensitive for S4. However, a large number of strains (18 of 43), which were resistant to S4, were also positive for the *fpvAI* gene (Table 3), raising the question about the cause of their insensitivity for S4. When we tested these strains by PCR, we found that all of them had the S4 immunity gene (results not shown), whereas all sensitive strains were negative for the S4 *imm* gene. This observation is in good agreement with the previous result showing that PAO1 (ΔS4+imm mutant) became sensitive to S4, whereas the wild-type PAO1 is not.

**Table 3:** Phenotypes of sensitivity or resistance to pyocin S4 of 43 *P. aeruginosa* isolates

	Ferripyoverdine receptor type					
	
	FpvAI		FpvAII		FpvAIII	
			
	Pyocin S4 sensitivity	Immunity gene presence	Pyocin S4 sensitivity	Immunity gene presence	Pyocin S4 sensitivity	Immunity gene presence
S4 resistant	18	+	13	−	6	−
S4 sensitive	6	−	0	−	0	−

**Figure 2:**
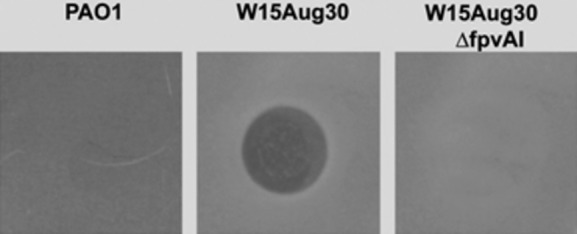
Effect of the inactivation of the type I *fpvA* gene in the pyocin S4-sensitive strain W15Aug30. Killing activity of pyocin S4 on: PAO1 wild-type (left), S4-sensitive strain W15Aug30 wild-type (middle), and the W15Aug30Δ*fpvA* (right) which became resistant due to the absence of receptor.

To further confirm the involvement of type I ferripyoverdine receptor in the uptake of pyocin S4, the *fpvAI* gene from the S4-sensitive strain W15Aug30 was inactivated by allelic replacements as described in Materials and Methods. As expected, the W15Aug30 Δ*fpvA*I mutant became fully resistant to pyocin S4 (Fig. 2). Therefore, we can confirm that pyocin S4, like S2, is using the FpvAI receptor to enter the cell.

**Figure 3:**
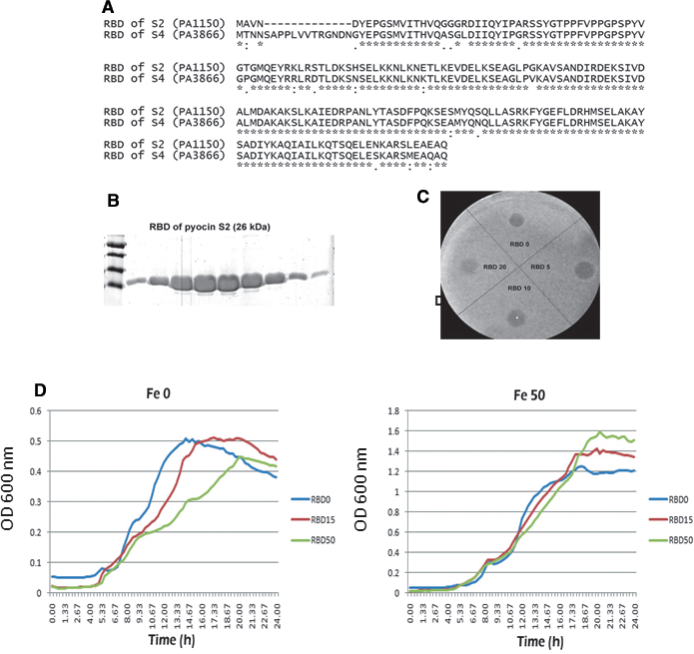
(A) Protein sequence alignment of the *N*-terminal receptor binding domains of pyocins S2 (PA1150) and S4 (PA3866) showing high similarity (77.29%). (B) SDS–PAGE analysis of different purification fractions of the *N*-terminal receptor binding domain (RBD) of the pyocin S2 protein (26 kDa). (C) Binding competition assay which is showing a decreased killing activity in the S4-sensitive strain W15Aug30 by spotting increasing amounts of RBD (0, 5, 10, 20 μg) together with a constant amount of pyocin S4. (D) Effect of the addition of increasing amounts (0, 15, 50 μg) of RBD on the growth of the insensitive *P. aeruginosa* PAO1 in CAA medium (Fe 0) or in CAA in the presence of 50 μmol / L FeCl_3_ (Fe 50).

Alignment of the amino acid sequences of S2 and S4 shows a good identity at the level of the N-terminal part of the two bacteriocins, as 178 residues of 211 of S4 are conserved in S2 (Fig. 3A), which is in good agreement with the fact that the N-terminal part of S-pyocins should correspond to the RBD (Michel-Briand and Baysse 2002).

### Binding competition assay

The *N*-terminal RBD of the pyocin S2 was cloned, and expressed in *E. coli*. The purified RBD migrated as a 26 kDa protein on SDS-PAGE and was stable (Fig. 3B). The purified S2 RBD was supplied in increasing concentrations together with a constant amount of pyocin S4. Then the mixture was spotted on a bacterial cell layer of the S4-sensitive strain W15Aug30 and incubated at 37°C for 24 h to see the effect of the addition of different concentrations of the RBD on growth inhibition caused by S4. The results (Fig. 3C) show a decreased killing activity of the W15Aug30-sensitive strain by S4 in the presence of increasing concentrations of RBD. The inhibitory effect of the RBD addition on the growth of the PAO1-insensitive strain in liquid culture was clearly observed when the cells were grown in CAA medium and in the absence of added iron, whereas no such inhibition was observed when the cells were grown in the same medium containing 50 μmol/L FeCl_3_ (Fig. 3D). This result confirms that the S2 RBD indeed competes with the pyocin S4 for the binding to the FpvAI receptor and that it probably inhibits the uptake of the ferripyoverdine via the same receptor.

### Activation of pyoverdine genes by the S2 RBD

**Figure 4:**
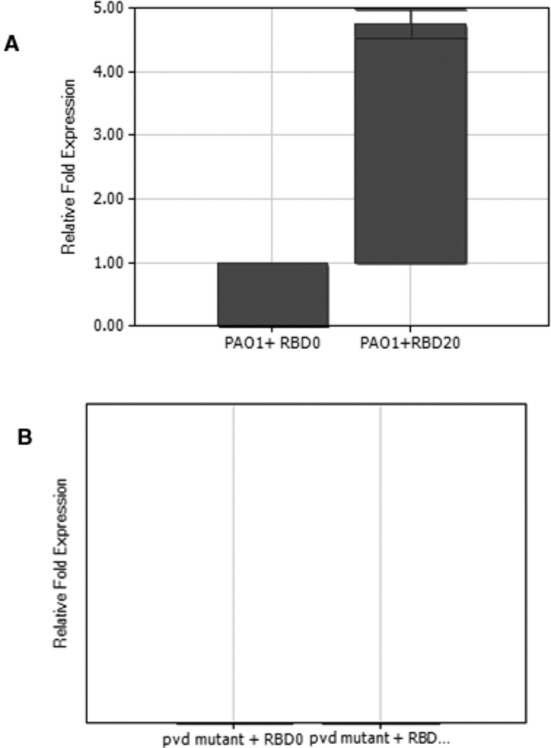
Effect of the addition of the S2 receptor binding domain (RBD) on the transcription of the *pvdA* pyoverdine biosynthesis gene in wild-type *P. aeruginosa* (A) and in the *pvdA* knock out mutant (B).

It has been established that pyoverdine in complex with iron binds to the FpvA receptor, which triggers a signaling cascade via the FpvR anti-sigma factor, causing the release of two extracellular sigma factors (ECF), PvdS and FpvI (Beare et al. 2003; Redly and Poole 2005; Spencer et al. 2008; Draper et al. 2011). PvdS sigma factor is needed for the transcription of pyoverdine biosynthesis genes, whereas FpvI is necessary for the expression of the *fpvA* receptor gene (Visca et al. 2007; Cornelis et al. 2009). We hypothesized that the RBD domain of S2 would interact with the FpvAI receptor, just like ferripyoverdine, triggering the liberation of PvdS, which, in turn, would increase the transcription of pyoverdine biosynthesis genes, such as *pvdA* (Leoni et al. 1996, 2000; Ambrosi et al. 2002). When the *P. aeruginosa* wild-type or *pvdA* mutant strains were grown in the presence of the S2 RBD, a clear up-regulation of the transcription of *pvdA* and *pvdS* was observed in the wild-type, whereas *pvdA* expression was, as expected, not detected in the *pvdA* pyoverdine-negative mutant (Fig. 4). This result suggests that the S2 RBD interacts with the same extracellular domains of FpvAI as type I pyoverdine, triggering the signaling cascade.

### Pyocin S4 killing activity

The BlastP program was used to search for proteins having a killing domain similar to the one of the pyocin S4. A high similarity alignment was found with Colicin E5 which has a tRNase activity (Ogawa et al. 2006; Cascales et al. 2007), confirming that S4 killing domain has a tRNAse activity as suggested by Parret and De Mot (2002).

## Conclusion

The results described here confirm that *P. aeruginosa* PAO1 has a functional immunity gene for pyocin S4. *Pseudomonas aeruginosa* PAO1 produces three S-type pyocins, S2, S4, and S5. Pyocin S5 has a different mode of action as it has a pore-forming activity (Ling et al. 2010) and the identification of its receptor is ongoing. Pyocins S2 and S3 have a highest killing activity when susceptible cells are grown under iron-limited conditions. That is because of their use of the ferripyoverdine receptor FpvA as a receptor (Smith et al. 1992; Duport et al. 1995; Baysse et al. 1999; de Chial et al. 2003; Denayer et al. 2007). Based on our results and the alignment of the RBDs of pyocins S2 and S4, we can conclude that the FpvA I receptor is indeed used by both the S4 and S2 pyocins to enter the cell and exert their killing activity. Furthermore, the S4-type pyocin showed a lower killing activity than pyocin S2, which could be due to differences in the killing domains, the DNAse activity of S2 being more efficient than the tRNAse domain of S4. Additionally, it has been shown that pyocin S2 almost completely inhibits lipid biosynthesis in susceptible cells, which, combined with the nuclease activity, could explain the higher toxicity of S2 (Okawa et al. 1975).

During this investigation, other interesting observations concerning the killing activity of S4 came out. Pyocin S4 killing domain indeed shares sequence and high structural homologies with the C-terminal domains of colicin E5, suggesting that it has a tRNase activity as previously predicted (Parret and De Mot 2002). Production of pyocins with different multifunctional killing domains could explain why *P. aeruginosa* is producing more than one type of pyocin targeting the same receptor. Further studies will be required to determine this point. Finally, we could demonstrate that the S2 RBD could be expressed and purified, and that it could inhibit the S4 killing in a concentration-dependent way. The same S2 RBD could induce the expression of the pyoverdine genes, just like pyoverdine does.
